# Prevalence of wasting and associated factors among 6 to 23 months old children in the Sahel Region of Burkina Faso

**DOI:** 10.11604/pamj.2019.34.164.19886

**Published:** 2019-11-26

**Authors:** Boyo Constant Paré, Désiré Lucien Dahourou, Ahmed Kabore, Adama Sana, Réné Kinda, Bertine Ouaro, Marie-Michelle Dahany, Hervé Hien, Nicolas Méda

**Affiliations:** 1 Département de Santé Publique, Unité de Formation et de Recherche en Sciences de la Santé, Université Ouaga I Pr Joseph Ki-Zerbo, Ouagadougou, Burkina Faso; 2 Institut de Recherche en Sciences de la Santé, Ouagadougou, Burkina Faso; 3 Centre Muraz, Institut National de Santé Publique, Bobo Dioulasso, Burkina Faso; 4 Centre National de Recherche et de Formation sur le Paludisme, Institut National de Santé Publique, Ouagadougou, Burkina Faso; 5 Direction de la Nutrition, Ministère de la Santé, Ouagadougou, Burkina Faso

**Keywords:** Nutritional status, acute malnutrition, risk factors, IYCF, National Nutrition Survey, Sahel region

## Abstract

**Introduction:**

Despite the efforts of nutrition stakeholders in Burkina Faso to improve infant and young child feeding (IYCF) practices, the country is still struggling to stem undernutrition. Wasting, or acute malnutrition, is the form of malnutrition that has the most harmful short-term consequences for children. The objective of our study was to estimate the prevalence of wasting in children aged 6-23 months in the Sahel region of Burkina Faso and to identify its associated factors.

**Methods:**

We conducted a secondary analysis of data from the 2015 National Nutrition Survey. The factors associated with wasting in the studied population were identified using a logistic regression.

**Results:**

A total of 956 children participated in the study. The prevalence of wasting was 25% (95% CI [22.28, 27.87]) in the Sahel region. Only 24.37% of children received a minimum meal frequency and 13.38% received a minimum dietary diversification the day before the survey. In the multivariate analysis, being male (aOR = 1.99; 95% CI [1.46, 2.72]), breastfeeding the day before the survey (aOR = 2.43; 95% CI [1.13, 5.22]), and having a history of illness (aOR = 2.32; 95% CI [1.67, 3.21]) significantly increased the risk of acute malnutrition.

**Conclusion:**

In 2015, the prevalence of wasting among children was high in the Sahel region and good IYCF practices were still inadequate. There is an urgent need to implement good IYCF practices and strengthen interventions to improve infant health in this region.

## Introduction

Malnutrition remains a public health concern for developing countries. The prevalence of different forms of malnutrition (acute malnutrition or wasting, stunting, and underweight) remains high, especially among children under 5 years of age [1]. Malnutrition weakens intellectual capacity, limits productivity in adulthood, increases vulnerability to certain diseases, and is the underlying cause of about 45% of all deaths in children under 5 years of age worldwide [2,3]. Wasting is the type of malnutrition that has the most harmful short-term consequences for children. Child wasting is defined by a weight-to-height ratio less than 2 standard deviations (SDs). It is driven by infectious diseases and an inadequate diet. It is a direct cause of mortality among children under 5 years of age [4]. The prevalence of wasting in children under 5 years old in 2015 was estimated at 7.4% worldwide and 8.9% in West Africa [5]. Despite the efforts of the government and nongovernmental organizations to improve infant and young child feeding (IYCF), wasting among children under 5 years of age remains a concern in Burkina Faso. To obtain reliable and up-to-date data on the nutritional status of children, Burkina Faso's Ministry of Health has annually conducted the National Nutrition Survey (NNS) since 2008. The NNS is carried out according to the Standardized Monitoring and Assessment of Relief Transition (SMART) methodology [6]. The surveys reported a steady decline from 11.3% in 2009 to 8.6% in 2014 in the national prevalence of wasting among children under 5 years of age [6-8]. However, the NNS carried out in November 2015, during the harvest period and the drop in the malaria peak, showed a reversal of this trend, with a prevalence of 10.4% [8]. The prevalence in the Sahel region, where the highest prevalence was reported (15.7% in 2015 versus 11.7% in 2014), was above the emergency threshold of 15% set by the World Health Organization (WHO) [7-9]. Nutrition stakeholders have sought explanations about the prevalence's peak in 2015. However the risk factors for wasting were still poorly documented in this region of Burkina Faso. The objective of our study was to describe the feeding practices of children aged 6-23 months old in the Sahel region and to identify the factors associated with wasting in 2015.

## Methods

**Type and period of study**: we conducted a secondary analysis of the 2015 NNS data. The 2015 NNS was a cross-sectional study based on SMART methodology with a national coverage [8,10]. It was carried out by Burkina Faso's Ministry of Health from November 16 to December 10, 2015. It focused on children under 5 years of age. Our study population included all children aged 6-23 months in the Sahel region who participated in the 2015 NNS.

**Data collected**: for our study, we extracted the children's anthropometric (weight and height), sociodemographic (age and sex), food, and clinical data (history of illness through the onset of fever or diarrhea 2 weeks before the survey and deworming with mebendazole). We also extracted mothers' education level. The weight measurement was conducted using uniscale weight scales (also called seca electronic weight scales) with an accuracy up to 100 g. The kilogram (kg) was the unit of measurement used. All of the children were weighed naked. Their heights were measured using a shorr measuring board (in wood) graduated in centimeters and accurate to the millimeter. The centimeter (cm) was the unit of measure. Children under 2 years of age were measured in the supine position. Wasting was defined according to WHO 2006 standards. A weight-to-height z-score (WHZ) less than <2 standard deviations (SDs) was considered as wasting (with severe wasting defined as a z-score less than <3 SDs). The data collected on infant feeding included the following: early initiation of breastfeeding (put to the breast within one hour of birth), the continuation of breastfeeding (breastfeeding the day before the survey), and the food groups consumed the day before (**group 1**: cereals, roots, and tubers; **group 2**: legumes and nuts; **group 3**: dairy products; **group 4**: meat products; **group 5**: eggs; **group 6**: fruits and vegetables rich in vitamin A; **group 7**: other fruits and vegetables). These nutritional data were used to calculate minimum dietary diversification (MDD), minimum meal frequency (MMF), and minimum acceptable diet (MAD) according to WHO guidelines related to assessment of IYCF practices [11]. 1) The MDD was considered good if children who consumed the day before the survey ate foods from at least 4 distinct food groups. 2) The MMF was considered good if the breastfed infant aged 6-8 months received at least 2 meals the day before the survey; the child aged 9-23 months should have received at least 3 meals. The MMF was considered good if non-breastfed children aged 6-23 months received at least 4 meals the day before the survey. 3) The MAD is a combination of the MDD and MMF. The calculation of this variable depends on the (non) continuation of breastfeeding. For breastfed children, MAD was considered good if the child received at least the MDD and MMF the day before the survey. For non-breastfed children, MAD was considered good if the child had received at least 2 milk feedings and foods from at least 4 distinct food groups (excluding milk feedings) the day preceding the survey.

**Statistical analysis**: the WHZ z-scores were calculated with the Emergency Nutrition Assessment for SMART 2011 software, according to the WHO 2006 standards, and the statistical analysis was performed using the Stata 12 software. Quantitative variables were described by their means and SDs. The qualitative variables were described by their numbers and percentages. The dependent variable was wasting. The independent variables were sociodemographic, clinical, and nutritional characteristics. The factors associated with wasting were studied using a logistic regression. The factors associated in the univariate analysis were selected at the 20% threshold for a multivariate model. The final model was obtained using a manual backward selection. MAD was excluded from multivariate analysis because of collinearity with MMF and MDD.

**Ethical and regulatory aspects**: during each NNS, the informed consent of the head of the household is needed to collect the children's data. Each child was assigned a unique number to maintain confidentiality during our study.

## Results

In the Sahel region, 973 children aged 6-23 months were included in the 2015 NNS. The anthropometric data were unavailable for 17 children, which led to their exclusion from our analyses. In total, we conducted analyses on 956 children.

**Sociodemographic and anthropometric characteristics of children 6-23 months of age in the Sahel region during the 2015 NNS in Burkina Faso**: the average age was 14.55 months (SD: 5.08) and 47.18% of the children were female. The average WHZ z-score was-1.22 (SD: 1.42). Wasting prevalence was 25% (95% CI [22.28, 27.87]) with 6.49% suffering from severe wasting (Table 1).

**Table 1:** Descriptive characteristics of the respondents

Variables	Frequence (n)	% or average ± SD
**Children age** (Month)	956	14.6 ± 5.1
**Children weight** (Kg)	956	8.0 ± 1.4
**Children length** (Cm)	956	72.7 ± 5.5
**z-score P / T**	956	-1.3 ± 1.4
**Wasting**	956	
No	717	75.0
Yes	239	25.0
Moderate	177	18.51
Severe	62	6.49
**Sociodemographic data**		
**Infant sex**	956	
Female	451	47.2
Male	505	52.8
**Infant age**	956	
6-11 months	351	36.7
12-23 months	605	63.3
**Mother Instruction Level**	955	
No	935	97.91
Primary	15	1.57
Secondary	5	0.52
University	0	0.00
**Diet and historiry of illness**		
**Early initiation of reastfeeding**	940	
<1 hour	479	50.9
**Breastfeeding**	955	
Yes	885	92.7
**Minimum dietary diversification**	956	
≥4	127	13.3
Minimum meal frequency*	840	
Good	556	66.2
**Minimum acceptable diet**	770	
Good	81	10.5
**History of illness last 2 weeks**	956	
Yes	554	57.9
**Deworming**	379	
Yes	319	84.2

% = Percent; SD = Standard Deviation

* Good= Breastfed children who received solid, semi-solid or soft foods the at least two times for children 6-8 months and three times for children 9-23 months the day before the survey, and non-breastfed children 6-23 months who received solid, semi-solid or soft foods or milk feeds at least four times per day.

**Food and disease of children aged 6-23 months**: during the 2015 NNS, the proportion of children being breastfed after 6 months of age was 92.67%, and the proportion who received the MMF the day before the survey was 66.19%. Only 13.38% of children had a good MDD score, and the MAD was only good in 10.42% of children. And 57.9% of children presented a history of illness (fever and or diarrhea) the last 2 weeks before the survey (Table 1).

**Factors associated with infant emaciation**: in a univariate analysis, deworming, male sex, continued breastfeeding, and history of illness in the last 2 weeks were significantly associated with acute malnutrition in children 6-23 months of age (Table 2). Age, sex, continued breastfeeding, history of illness, and deworming were associated with acute malnutrition at a threshold of 20% and were selected in the multivariate model.

**Table 2:** Factors associated with wasting of children 6-23 months, univariate analysis

	Wasting			
Independent variables	n / N (%)	OR	95% CI	p value
**Age**				
6-11mois	100/351 (28.5)	1		
12-23mois	139/605 (22.9)	0.74	0.55, 1.00	0.05
**Sex**				
Female	83/451 (18.4)	1		
Male	156/505 (30.9)	1.98	1.46, 2.68	˂0,001 *
**Level of instruction of the mother**				0.21
No	236/935 (25.2)	1		
Primary	3/15 (20.0)	0.74	0.20, 2.64	0.64
Secondary	0/5 (0.0)	-	-	-
University	-	-	-	-
**Early initiation of breastfeeding**				0.52
<1 hour	115/479 (24.0)	1		
**Breastfeeding**				
No	8/70 (11.4)	1		
Yes	231/885 (26.1)	2.73	1.29, 5.80	0,009 *
**Minimum meal frequency**				
Bad	74/284 (26.1)	1		
Good	135/556 (24.3)	0.90	0.65, 1.26	0.57
**Minimum dietary diversification**				
Bad	212/829 (25.6)	1		
Good	27/127 (21.3)	0.78	0.49, 1.23	0.29
**Minimum acceptable diet**				
Bad	185/689 (26.7)	1		
Good	16/81 (19.8)	0.67	0.37, 1.18	0.17
**History of illness last 2 weeks**				
No	65/402 (16.2)	1		
Yes	174/554 (31.4)	2.37	1.72, 3.27	˂ 0.001 *
**Deworming**				
No	7/60 (11.7)	1		
Yes	68/319 (21.3)	0.48	0.21, 1.12	0.09

n = number of wasted children; N =Total; OR = Odds ratio; CI_95%_ = 95% confidence interval; p = degree of significance

* : Value with a significant p (below the threshold of 0.05)

**Multivariable analysis of factors associated with infant emaciation**: in the adjusted analysis, 3 variables were independently associated with acute malnutrition: sex, breastfeeding, and a history of illness. The odds of acute malnutrition were significantly higher among breastfed children (aOR = 2.43; 95% CI [1.13, 5.22]), male children (aOR = 1.99; 95% CI [1.46, 2.72]), and children with a history of illness in the 2 weeks preceding the survey (aOR = 2.32; 95% CI [1.67, 3.21]; Table 3). There was no interaction between the variables.

**Table 3:** Factors associated with acute malnutrition of children 6-23 months, multivariate analysis

Independent variables	Full model				Final model		
	n/N (%)	OR	95% CI	p value	aOR ^*^	95% CI	p value
**Age**							
6-11mois	100/351 (28.5)	**1**					
12-23mois	139/605 (22.9)	0.10	0.01, 1.26	0.07			
**Sex**							
Female	83/451 (18.4)	1			**1**		
Male	156/505 (30.9)	2.78	1.57, 4.92	**˂0,001**	1.99	1.46, 2.72	˂0,001 *
**Breastfeeding**							
No	8/70 (11.4)	1					
Yes	231/885 (26.1)	3.32	1.13, 9.62	**0.02**	2.43	1.13, 5.22	0.02 *
**History of illness last 2 weeks**							
No	65/402 (16.2)	1			1		
Yes	174/554 (31.4)	2.44	1.35, 4.41	**0,004**	2.32	1.67, 3.21	˂0,001 *
**Deworming**							
No	7/60 (11.7)	1					
Yes	68/319 (21.3)	0.42	0.17, 1.01	0.05			

n = number of wasted children; N =Total; aOR= adjusted Odds Ratio; CI _95 %_ = 95% confidence interval; p = degree of significance

* : Associated value after adjustment in the final multi-variate logistic model

Adjustment in the final model was done on sex, continuation of breastfeeding and illness (diarrhea and fever) the last 2 weeks before the survey. There was no interaction.

## Discussion

Our study showed a high prevalence (25%) of wasting in children aged 6-23 months in the Sahel region of Burkina Faso. Food diversification was not good for the majority of children in this region. The risk of acute malnutrition was significantly higher among male children, children who breastfed the day before the survey, and children with a history of disease in the 2 weeks preceding the survey. Even though the 2015 survey occurred during the harvest period, which also corresponds to the drop of malaria cases among children [12,13], we recorded a high prevalence of acute malnutrition in children 6-23 months in the Sahel region. It was above the emergency threshold defined by the WHO. The high sensitivity of wasting to changes from one period to another might explain the variation in prevalence. Indeed, wasting is often associated with short-term factors such as seasonal variations in food availability, acute food shortages, changes in socioeconomic policies, and diseases emerging beyond the expected level [14-17]. The sociopolitical context, which constitutes a fundamental determinant of malnutrition [18], was also tense between 2014 and 2015 in Burkina Faso [19]. Between September 2014 and September 2015, the country experienced a significant deterioration of the sociopolitical environment, contributing to the decrease in the production of cereals, food crops, and cash crops during the 2014-2015 and 2015-2016 crop years [20,21]. Additionally, the 2015-2016 crop year drought touched 43.5% of the cultivated land in the Sahel region, creating a cereal deficit [21]. As a result, food was mainly imported in this region that is characterized by poverty and pastoralism. Poor access to cereals, the main source of food in the region, and a high prevalence of febrile and diarrheal diseases in children [22,23] may explain the high prevalence of wasting in this region.

In the Sahel region, the majority (92.67%) of children aged 6-23 months were breastfed. Continued breastfeeding significantly increased the risk of emaciation. This result shows that the period of introduction for complementary food after the first 6 months of the infant's life is crucial in the infant's nutrition [24,25]. In fact, the relationship between the continuation of breastfeeding and the infant's height-weight index depends on the quality and the amount of additional food received by the child [26]. However, the MDD and MAD were good in less than 15% of children. This result may be explained by the mothers' misconception about the need to introduce complementary foods at the age of 6 months and by the low access to quality food [25]. The promotion of continued breastfeeding should go hand in hand with improved access to complementary foods and mothers' education about good infant feeding practices. We showed that a history of illness (through diarrhea and/or fever) 2 weeks before the survey significantly increased the risk of emaciation. These results could be explained by inadequate hygiene practices during breastfeeding and during infant feeding. In the Sahel region, in the pastoral zone specifically, the current practice is to feed children with fresh, unpasteurized animal milk. This practice may transmit pathogens to the infant, leading to diarrheal diseases, which have a direct effect on the infant's nutritional status [24]. Additionally, the population of the arid Sahel region has difficulties accessing drinking water. Thus, a study carried out in Bamako, Mali (also in the Sahel region), demonstrated the association between hygiene practices, access to drinking water, and child malnutrition [17].

In our study, we did not show a statistically significant association between the indicators of IYCF (MDD and MMF) and wasting. This lack of association has been reported by previous studies [25,27-31]. This result may be explained by the high frequency of misclassifying an adequate diet when administering the IYCF questionnaire. The simplicity of the WHO IYCF indicators might simplify the complexity inherent in children's complementary feeding. Thus, the IYCF indicators may miss some contextual facts and therefore lack specificity [27]. For example, an infant received the equivalent of a teaspoon of rice with a palm oil sauce containing a small piece of fish. Is it sufficient to consider this meal as one containing 4 food groups; cereals, nuts, vegetables rich in vitamin A, and meat products consumed by the child? If that is the case according to the respondent, the indicators will not properly express the quantity and quality of the food consumed and will lack specificity. There is a need to develop new, more specific indicators adapted to the African context to assess the quality and quantity of infant feeding. Our study has some limitations. The evaluation of the IYCF indicators was made from the declarations of mothers of children, which could have led to an information bias and created a misclassification of the children. Some important variables to explain the nutritional status of children (e.g. food availability in the household, the average monthly household income, household size, and the number of infants' brothers and sisters) were not collected in the 2015 NNS. We have considered the variable level of education as a proxy for socioeconomic data in order to offset the lack of socioeconomic and demographic variables. Some data are missing for the variable of interest (17/973 = 1.74%). We have assumed these data were missing randomly, and we conducted an analysis of the available data. In spite of these limitations, our study, which focused on a large-scale representative sample of children from the Sahel region of Burkina Faso, enabled us to identify factors associated with the emaciation of children.

## Conclusion

Our study showed that during the 2015 NNS, the prevalence of wasting in children aged 6-23 months was higher in the Sahel region than in others. Additionally, it showed that several factors were associated with the growth of children. Our results suggest that despite the IYCF promotion programs, good IYCF practices were not sufficient there. There is an urgent need to implement good IYCF practices and strengthen interventions to improve infant health in this region. It is important to evaluate the real effectiveness of IYCF awareness activities on mothers' practices when feeding their children.

### What is known about this topic

- Wasting prevalence is often high in the Sahel region of Burkina Faso;
- Immediate causes of malnutrition are diet and disease.

### What this study adds

- Breastfeeding and mothers' adequate hygiene during this phase are still insufficient and need improvement.
- It is important to evaluate and strengthen the promotion of IYCF practices in this location.

## Competing interests

The authors declare no competing interests.
